# Functional Pathway Analysis Using SCNP of *FLT3* Receptor Pathway Deregulation in AML Provides Prognostic Information Independent from Mutational Status

**DOI:** 10.1371/journal.pone.0056714

**Published:** 2013-02-19

**Authors:** Alessandra Cesano, Santosh Putta, David B. Rosen, Aileen C. Cohen, Urte Gayko, Kavita Mathi, John Woronicz, Rachael E. Hawtin, Larry Cripe, Zhuoxin Sun, Martin S. Tallman, Elisabeth Paietta

**Affiliations:** 1 Nodality, Inc., South San Francisco, California, United States of America; 2 Eastern Cooperative Oncology Group (ECOG), Boston, Massachusetts, United States of America; 3 Indiana University Simon Cancer Center, Indianapolis, Indiana, United States of America; 4 Dana-Farber Cancer Institute, Boston, Massachusetts, United States of America; 5 Memorial Sloan-Kettering Cancer Center, New York, New York, United States of America; 6 Montefiore Medical Center North Division, Bronx, New York, United States of America; UT MD Anderson Cancer Center, United States of America

## Abstract

FMS-like tyrosine kinase 3 receptor (*FLT3*) internal tandem duplication (ITD) mutations result in constitutive activation of this receptor and have been shown to increase the risk of relapse in patients with acute myeloid leukemia (AML); however, substantial heterogeneity in clinical outcomes still exists within both the ITD mutated and unmutated AML subgroups, suggesting alternative mechanisms of disease relapse not accounted by *FLT3* mutational status. Single cell network profiling (SCNP) is a multiparametric flow cytometry based assay that simultaneously measures, in a quantitative fashion and at the single cell level, both extracellular surface marker levels and changes in intracellular signaling proteins in response to extracellular modulators. We previously reported an initial characterization of FLT3 ITD-mediated signaling using SCNP. Herein SCNP was applied sequentially to two separate cohorts of samples collected from elderly AML patients at diagnosis. In the first (training) study, AML samples carrying unmutated, wild-type *FLT3* (*FLT3* WT) displayed a wide range of induced signaling, with a fraction having signaling profiles comparable to *FLT3* ITD AML samples. Conversely, the *FLT3* ITD AML samples displayed more homogeneous induced signaling, with the exception of patients with low (<40%) mutational load, which had profiles comparable to *FLT3* WT AML samples. This observation was then confirmed in an independent (verification) cohort. Data from the second cohort were also used to assess the association between SCNP data and disease-free survival (DFS) in the context of *FLT3* and nucleophosmin (*NPM1*) mutational status among patients who achieved complete remission (CR) to induction chemotherapy. The combination of SCNP read outs together with *FLT3 and NPM1* molecular status improved the DFS prediction accuracy of the latter. Taken together, these results emphasize the value of comprehensive functional assessment of biologically relevant signaling pathways in AML as a basis for the development of highly predictive tests for guidance of post-remission therapy.

## Introduction

FMS-like tyrosine kinase 3 receptor *(FLT3)* receptor mutations are among the most common somatic mutations in acute myeloid leukemia (AML), with *FLT3* internal tandem duplications (ITDs) occurring in 20–35% of adult AML [1], [2], [3], [4], [5], [6], [7] and ∼5–15% of pediatric AML [8], [9], [10]. While the presence of *FLT3* ITD is not predictive of outcome to induction chemotherapy, it has consistently been shown to be associated with significantly shorter disease-free (DFS) and relapse-free survival [11]. The length of the DNA insertion that constitutes the ITD varies among patient leukemia samples, with those that contain a high level of mutated to WT DNA ratio faring worse [2], [12], [13].

Nucleophosmin (*NPM1*) is a nucleolar phosphoprotein that is mutated in 25–35% of patients with AML and is associated with good prognosis, including in patients of advanced age [14], [15], [16]. Molecular characterization of the *FLT3* receptor and *NPM1* mutational status in cytogenetically normal AML has been incorporated into clinical guidelines based on correlations of these markers with overall survival [17]. Patients with *NPM1* mutation but *FLT3* WT are thought to be of good prognostic risk, while *FLT3* ITD but *NPM1* WT patients are at high risk for disease relapse and should be considered for stem cell transplant (SCT) in first complete remission (CR). AML with *NPM1* WT*/FLT3* WT or carrying both *NPM1* mutation and *FLT3* ITD are considered to have intermediate-risk disease [17]. However, clinical heterogeneity remains in these cytogenetically and molecularly defined AML subgroups [18].

*FLT3* receptor mutations negatively affect outcome in AML, and kinase activity is already being targeted with kinase inhibitors in the clinic. Downstream targets of FLT3 activation involve STAT5, PI3-kinase (PI3K)/AKT and the RAS/RAF/ERK kinase signal transduction pathways which ultimately affect cell survival and proliferation [19], [20], [21], [22], [23], [24], [25], [26].

Previously, we reported the use of SCNP to functionally characterize FLT3 signaling in healthy bone marrow (BM) and samples obtained from adult patients (<60 years) with AML [27]. AML carrying the *FLT3* ITD mutation demonstrated decreased FLT3 ligand (FLT3L)-induced activation of the PI3K and RAS/RAF/ERK pathways, decreased IL-27-induced activation of the JAK/STAT pathway, and heightened apoptotic responses to agents inducing DNA damage [27] compared to healthy BM samples. In the same study, we also showed a relatively narrow range of signaling responses in *FLT3* ITD samples compared to *FLT3* WT AMLs suggesting that the latter represented a more heterogeneous and biologically distinct group of leukemias. On the basis of these observations we hypothesize that assessing patient samples for the presence of FLT3 signaling deregulation may provide clinically relevant prognostic information that is independent of AML mutational status.

**Figure 1:**
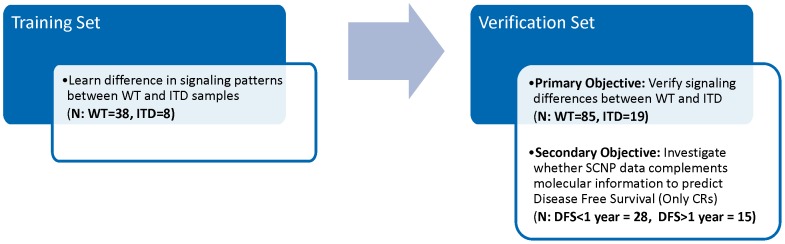
Study Design Diagram. Influence of *FLT3* ITD mutation status on functional signaling was studied in two independent data sets; observations made in the first set (training, N = 46) were verified in the second set (Verification, N = 104).

In the current study this hypothesis was tested and confirmed in two sequential cohorts (Fig. 1) using diagnostic samples from elderly patients (≥60 years) with AML who participated in Eastern Oncology Cooperative Group (ECOG) 3999 [28] or 3993 [29] AML trials, which are comparable for the purpose of the analyses performed in the current study.

## Materials and Methods

### Ethics Statement

In accordance with the Declaration of Helsinki, all patients provided written informed consent for the collection and use of their samples for research purposes. Institutional Review Board approval was obtained from Independent Review Consulting, Inc. (Approval No. 09068-01) on August 31, 2009. Clinical data were de-identified in compliance with Health Insurance Portability and Accountability Act regulations.

### Patient and sample characteristics

The first (training) study consisted of 46 cryopreserved bone marrow mononuclear cell (BMMC) samples collected at the time of diagnosis from patients ≥60 years of age with morphologically and immunophenotypically confirmed AML, enrolled in Phase 3 trial E3999. Samples included 38 *FLT3* WT and 8 *FLT3* ITD AML. The second (verification) study consisted of 104 cryopreserved BMMC samples collected from patients ≥60 years old enrolled in Phase 3 trials E3999 or E3993 for whom ITD mutational status (including % mutational load), response and DFS were available (Fig. 1). These included 85 *FLT3* WT and 19 *FLT3* ITD AML, 29 and 10 of which, respectively, were collected from patients who achieved complete remission (CR). Both trials excluded *PML/RARα* positive acute promyelocytic leukemia.

Samples were collected prior to the initiation of anthracycline/cytarabine (Ara-C)-based induction chemotherapy and BMMC were cryopreserved in the ECOG Leukemia Tissue Bank until release for this study. The sample sets used for the present study represent a subset of the subjects enrolled on the ECOG studies which were selected based on the availability of stored aliquots of BMMC samples. In order to be included in the experimental analysis, BMMC specimens had to contain a minimum of 2 million viable cells post-thaw.

Based on data obtained in the training study, prespecified sample inclusion criteria for the verification study were modified to include sample quality elements and require that the percentage of leukemic (identified using CD45 and side scatter) cells that were cleaved PARP (cPARP) negative (non-apoptotic leukemic cells) was greater than 25% at the 15 minute time point (minimum cell health criteria, see Flow cytometry data acquisition below for further explanation) and that molecular assay data, including percentage of mutational load for the *FLT3* ITD samples, was also available.

Healthy BMMC (n = 6) were obtained from patients at the time of hip replacement surgery from Williamson Medical Center (TN). These samples (5 male, 1 female) were from predominately Caucasian donors who ranged in age from 60–87 years, (median = 71 years).

### SCNP assay

SCNP assays were performed as described previously [27], [30], [31]. Thawed cells were stained with Aqua Viability Dye to distinguish non-viable cells. For apoptosis assays, cells were incubated with cytotoxic drugs for 6 hours (staurosporine) or 24 hours (etoposide or Ara-C and daunorubicin) and re-stained with Aqua Viability Dye. For all other assays, cells were incubated with modulators (Table S1A) at 37°C for 5–15 minutes. After exposure to modulators, cells were fixed and permeabilized as previously reported [27], [30], [31]. Subsequently, cells were stained with cocktails of fluorochrome-conjugated antibodies (Table S1B), against 2 to 5 phenotypic markers for cell population gating and up to 3 antibodies against intracellular signaling molecules for an 8-color flow cytometry assay. Isotype controls (for surface marker antibodies) or phosphopeptide blocking (for antibodies against intracellular signaling molecules) were included as part of the characterization of each of the antibodies used in the assay. Due to insufficient number of cells for one sample in the training study SCNP data could not be collected for all conditions; as a result data is available for either 7 or 8 *FLT3* ITD donors depending on the condition tested.

### Signaling pathways evaluated

Modulated cell signaling pathways previously shown to be associated with *FLT3* ITD mutational status were examined [27]. These included: a) SCF and FLT3L-mediated activation of the PI3K/AKT or RAS/RAF/ERK pathway which each contributes to the survival and proliferation of AML blast cells [32], [33], [34] and is important for maintaining the hematopoietic stem cell pool [35], [36]; b) G-CSF and IL-27-mediated activation of the JAK/STAT pathway, whose proliferative and survival properties likely plays a central role in AML leukemogenesis as well as clinical resistance/refractoriness to genotoxic agents [37], [38], [39], [40]; and c) staurosporine, etoposide, or Ara-C/daunorubicin-mediated induction of DNA damage and apoptosis pathways after *in vitro* exposure (transformed cells can evade apoptosis by activating survival pathways and/or disabling apoptotic pathways) [41].

### Flow cytometry data acquisition

Flow cytometry data were acquired on an LSR II and/or CANTO II flow cytometer using the FACS DIVA software (BD Biosciences, San Jose, CA) and analyzed with WinList (Verity House Software, Topsham, ME). Dead cells and debris were excluded by forward scatter (cell size), side scatter (granularity), and Amine Aqua Viability Dye measurement. Leukemic cells and normal myeloblasts were identified as cells that fit the CD45 versus right-angle light-scatter characteristics of myeloblasts [42]. Additional phenotypic markers (CD34, CD11b, CD15) were used to clarify the leukemic population. Non-apoptotic leukemic cells were identified as leukemic cells negative for cPARP, an activated caspase target. Previous data demonstrated that SCNP signaling responses are robust in non-apoptotic cPARP negative cells but not in apoptotic cPARP positive cells [43], and that AML samples with less than 25% cPARP negative leukemic cells are generally not analyzable for signaling responses [44]. Thus, our current analyses include a “sample quality” cutoff of at least 25% cPARP negative leukemic cells and a measurement of SCNP signaling responses limited (by gating) to cPARP negative leukemic cells to control for sample quality differences among individual AML samples. Of note, the percentage of non-apoptotic leukemic cells was not statistically different between *FLT3* WT and any of the *FLT3* ITD groups both in variance (using Levene Test) or magnitude (using student's t-test) (data not shown). Healthy bone marrow myeloblasts (BMMb) were identified in BMMC samples using right-angle light scatter and surface markers such as CD45 and CD34.

### Metrics

In SCNP assay terminology, a “signaling node” is used to refer to a proteomic readout in the presence or absence of a specific modulator. For example, the response to FLT3L treatment can be measured using p-AKT as a readout. That signaling node is designated “FLT3L→p-AKT”. The normalized assay readouts for surface and intracellular markers are evaluated using metrics that are applied to interpret the functionality and biology of each signaling node. They are referenced following the node e.g. “FLT3L→p-AKT | Fold”, “G-CSF→p-STAT5 | Total” and are defined in detail below.

Median fluorescence intensity (MFI) was computed for each node from cell fluorescence intensity levels. Equivalent Number of Reference Fluorophores (ERF) [45], [46], [47], a transformed value of the MFI value, was computed using a calibration line determined by fitting observations of a standardized set of 8-peak rainbow beads for all fluorescent channels (Spherotech Libertyville, IL; Cat. No. RFP-30-5A) to standard values assigned by the manufacturer.

The ERF values were then used to compute a variety of metrics to quantify functional changes in signaling proteins as follows (see also Fig. S1): A) Basal ERF (“Basal”): defined as log_2_(ERF_Unmodulated_/ERF_Autofluorescence_) to measure basal levels of signaling in the resting, unmodulated cells; B) Fold Change ERF (“Fold”): defined as log_2_(ERF_Modulated_/ERF_Unmodulated_) to quantify the responsiveness of a protein or pathway to a specific modulator; C) Total Phospho ERF (“Total”): defined as log_2_(ERF_Modulated_/ERF_Autofluorescence_) to assess the magnitude of total activated protein after modulation; D) U_u_ metric, which is the Mann-Whitney U statistic comparing the fluorescence intensity values of the modulated and unmodulated cells scaled to the unit interval (0,1), was used to assess the proportion of cells which showed modulated signal; E) U_a_ metric, (comparing ranking of the antibody stained cells in the modulated state vs. autofluorescence) was used to quantify the percentage of cells showing signal induction post-modulation. Finally, for surface markers, the Percent Positive (“PercentPos”) was used to quantify the frequency of cells positive for a surface marker relative to a control antibody. For measures of induced signaling, one metric per node was generally used as Fold and U_u_ metrics gave highly concordant results (Average Pearson R: 0.92 for the nodes studied, data not shown). For short term signaling, the the Fold metric wastipically used based on the potentially higher dynamic range of this metric with the exception of p-AKT intracellular readout (measured in the Alexa Fluor AF647 channel) which displayed low (near zero in unmodulated wells) absolute median intensity values which contribute to less reproducible Fold metrics. A rank based metric U_u_ which approximates the proportion of cells that were induced was found (based on reproducibility data) to be more robust in such situations. Similarly, apoptosis readouts were measured using rank based U_u_ or U_a_ metrics which approximate the proportion of cells undergoing apoptosis and were previously found to be more stable than metrics based on the absolute levels of cPARP staining or Fold metrics (data not shown).

### Analysis of *FLT3* receptor mutational status and load

An aliquot of cells was taken from each sample to isolate DNA and RNA using TRIzol Reagent (Invitrogen) as described by the manufacturer. For the analysis of *FLT3* ITD, PCR was performed on 500 ng genomic DNA using published primers 11F and 12R to identify ITD insertions in the JM and TK1 domains [48] using AmpliTaq Gold DNA polymerase (Applied Biosystems). *FLT3* ITD mutational load was determined using densitometry analysis software (Image J) quantifying the percentage of *FLT3* ITD mutant PCR signal intensity from DNA electrophoresis gels.

### Analysis of *NPM1* mutational status

Real-time quantitative PCR assays for *NPM1* mutations A and B were performed to analyze the *NPM1* mutational status. RQ-PCR was performed on 100 ng cDNA using published Taqman primers for mutation A (cNPM-F and cNPMmutA-R) or mutation B (cNPM-F and cNPM-mutB-R) and a common probe (c.Probe), as described [49]. A 35 cycle threshold cutoff was used to identify samples positive for the *NPM1* mutation.

### Principle component analysis

Principle component analysis [50] was performed using the R Software package (version 2.13.0) to combine multiple nodes (dimensions) belonging to FLT3L, IL-27-induced signaling and etoposide-induced apoptosis measured by the U_u_ metric. The U_u_ metric gives each node an equivalent scale/range of signaling (from 0 to 1) which is preferable for PCA analysis. The first two principal components were then used to visually compare *FLT3* ITD, *FLT3* WT and Healthy BMMb sub-groups.

### Statistical analysis: Verification of decrease in range of FLT3L-induced pS6 levels in *FLT3* ITD samples with increase in mutational load

Four sub-groups of *FLT3* ITD samples were pre-defined and specified prior to unblinding in the second study; those with any percentage of mutation (mutational load) (*FLT3* ITD+), those with mutational load greater or equal to 30% (*FLT3* ITD+ 30), 40% (*FLT3* ITD+ 40) or 50% (*FLT3* ITD+ 50). The variance in the range of FLT3L-induced pS6 levels observed in each of these sub-groups and *FLT3* WT samples was computed by using the median as the central measure. Levene's test for the equality of variance was applied to compare each of the *FLT3* ITD sub-groups with the *FLT3* WT group using the method ‘levene.test’ from the package ‘lawstat’ in the R software package (version 2.13.0). The p-value from the Levene's test is reported for each of the comparisons.

### Statistical analysis: Analysis of association between SCNP data and DFS

Disease-free survival (DFS) was defined as the number of days from CR until relapse, death from any cause, or last contact and therefore is only applicable to patients who achieved CR.

All DFS modeling was performed as a continuous variable. Cox-proportional hazards regression was used to develop models for DFS using the package ‘survival’ in the R Software package (version 2.13.0). Due to the small size of the data set, the complexity of these models was limited to combining only one SCNP node at a time with either the *FLT3* ITD mutational status or molecular classification along with an interaction term. Molecular classification combined *FLT3* and *NPM1* molecular status into a single coding of 1 for those patients with good prognosis (*FLT3* WT and *NPM1* MUT), 2 for those with intermediate (*FLT3* WT and *NPM1* WT or *FLT3* ITD and *NPM1* MUT) and 3 for those with poor prognosis (*FLT3* ITD and *NPM1* WT). Additional modeling of DFS was also performed only among the patients with CR and *FLT3* WT, where two SCNP nodes (each of which represented a different pathway) were combined to model DFS. The p-value for the model (Wald test) and the p-value (slope = 0) for each of the components of the model are reported. In addition, within the CR and *FLT3* WT group, Cox-proportional hazard models for DFS with two SCNP nodes were built with *NPM1* mutation status or cytogenetics as an additional covariate to examine if the SCNP nodes remained significantly associated with DFS. This data set contained several patients with missing cytogenetics data; in particular among the CR group 5 patients were coded by ECOG as Unknown cytogenetics. For modeling purposes, patients with missing cytogenetics were considered intermediate-risk, as is standard in clinical practice[51], [52]. A cytogenetics risk value was assigned to each patient: Favorable risk assigned 1; Intermediate, Unknown risk assigned 2; and Unfavorable risk assigned 3 to closely approximate risk assessments in a clinical setting. The models are also presented visually using Kaplan-Meier curves with the patients stratified by the model fit (i.e. applying the Cox model to the same data set); patients with a hazard ratio greater than 1.0 were plotted on the a curve labeled *Model+* and those with less than 1.0 were plotted on the curve labeled *Model−*.

## Results

### Clinical characteristics

The clinical and demographic characteristics of the patients from both the training and verification studies are shown in Table 1. Studies were matched across clinical variables with the exception of a predominance of secondary AML in the training study (66% training vs. 29% test). None of the variables examined in either study, including age, gender, race, white blood cell count or blast percent at presentation, or *NPM1* mutational status were associated with *FLT3* ITD mutational status (Table 1).

**Table 1:** Clinical characteristics for training and verification sets.

		Study 1: training				Study 2: verification			
Variable	Categories	*FLT3* ITD− n = 38	*FLT3* ITD+ n = 8	All (%) n = 46	*P*	*FLT3* ITD− n = 85	*FLT3* ITD+ n = 19	All (%) n = 104	*P*
Age, years	Median	69.5	70	70	0.73	69	66	68	0.15
	Range	61–81	61–74	61–81		60–83	61–79	60–83	
Gender	Male	23	5	28 (60.9)	1.00	46	9	55 (53)	0.62
	Female	15	3	18 (39.1)		39	10	49 (47)	
Cytogenetic risk	Favorable	0	0	0 (0)	0.24	2	1	3 (3)	0.31
	Intermediate	25	8	33 (71.7)		28	5	33 (31.7)	
	Unfavorable	11	0	11 (23.9)		26	3	29 (28)	
	Missing/unk	2	0	2 (4.3)		29	10	39 (37.5)	
Race	White	36	8	44 (95.7)	1	81	19	100 (96)	1
	Black	2	0	2 (4.3)		4	0	4 (4)	
Disease	De novo	12	2	**14 (30.4)**	1.00	56	14	**70 (67)**	0.9
	Secondary	24	6	**30 (65.2)**		25	5	**30 (29)**	
	RAEB-t	2	0	2 (4.3)		3	0	3 (3)	
	RAEB	0	0	0 (0)		1	0	1 (1)	
WBC	30 000 or less	21	4	25 (54.3)	1	56	11	67 (64)	0.59
	Greater than 30 000	17	4	21 (45.7)		29	8	37 (36)	
	Median (×10∧3)	23.35	26.9	23.35	0.82	15.3	26.8	16	0.31
	Range (×10∧3)	1.8–158.7	3.4–91.1	1.8–158.7		1.4–120.2	5.4–97.7	1.4–120.2	
*NPM1*-mutated AML	Yes	10	4	14 (30.4)	0.48	24	8	32 (31)	0.28
	No	26	4	30 (65.2)		61	11	72 (69)	
	Unknown	2	0	2 (4.3)		0	0	0 (0)	

Bold text indicates fields that are significantly different between the training and verification sets.

WBC indicates white blood cell; *NPM1*, nucleophosmin 1; RAEB-t, refractory anemia with excess blasts in transformation; and RAEB, refractory anemia with excess blasts.

### Training study

#### *FLT3* ITD samples show lower FLT3L-induced PI3K, RAS, and JAK signaling

**Figure 2:**
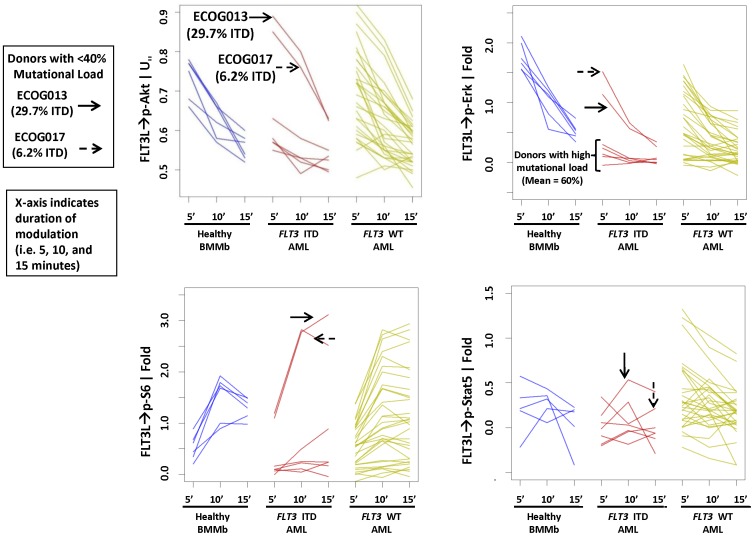
Muted FLT3L-induced signaling in *FLT3* ITD samples. (A) *FLT3* ITD samples demonstrate lower FLT3L-induced PI3K, RAS and STAT signaling. Time-course of FLT3L-induced signaling of p-S6 (lower left), p-ERK (upper right), p-AKT (upper left), and p-STAT5 (lower right) at 5, 10 and 15 min time points in healthy bone marrow myeloblasts (BMMb) (left), and leukemic blasts from AML donors with (middle) or without (right) *FLT3* ITD mutation. Donors with low mutational load (<40%) are identified with an arrow.

Similar to previous findings [27], FLT3 receptor surface protein levels did not differ between *FLT3* WT and *FLT3* ITD samples (data not shown). Incubation of cells with FLT3L resulted in the phosphorylation (p) of S6, ERK, AKT, and STAT5 at 5, 10 and 15 minute time-points in healthy BMMb and leukemic blasts from AML with or without *FLT3* ITD mutation. Induced signaling in the *FLT3* ITD AML samples (N = 8) was muted except in patients with low (<40%) mutational load (Fig. 2). In contrast, signaling in *FLT3* WT AML samples (N = 38) showed a high level of heterogeneity, with some samples showing patterns of signaling similar to that of healthy BM and *FLT3* ITD AML (Fig. 2). Furthermore, in the training set it was observed that FLT3L-induced signaling in *FLT3* ITD AML was muted when the *FLT3* ITD mutational load is high for multiple readouts at all timepoints examined (Fig. 2);

#### *FLT3* ITD samples show high levels of apoptosis in response to drugs

**Figure 3:**
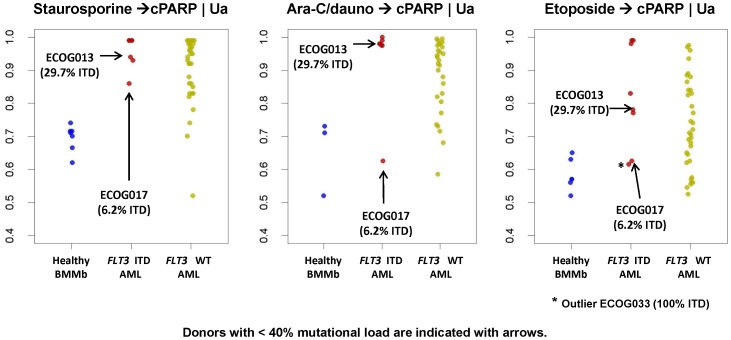
*In vitro* apoptosis responses in *FLT3* ITD samples. Staurosporine→cPARP U_a_ metric (left graph), Ara-C/Daunorubicin→cPARP U_a_ metric (middle graph), and etoposide→cPARP U_a_ metric (right graph) for healthy (left), *FLT3* ITD (middle) and *FLT3* WT (right) bone marrow. Samples with low mutational load (<40%) are identified with an arrow.

Apoptosis was evaluated as the proportion of cells that accumulated above the background of cPARP using the U_a_ metric (Fig. 3). *FLT3* WT samples exhibited a broad range of apoptotic responses when incubated with either Staurosporine or Ara-C/Daunorubicin. Conversely, *FLT3* ITD containing samples behaved more uniformly and showed robust levels of cPARP after Staurosporine treatment, consistent with previous findings [27]. All *FLT3* ITD samples exhibited high levels of apoptosis after treatment with Ara-C/Daunorubicin except for a single sample that contained low (6.2%) mutational load. Similar results were seen in response to etoposide although an additional sample in the *FLT3* ITD group (ECOG-033, 100% ITD) displayed a low apoptosis response that was not explained by low mutational load (Fig. 3). While this trend of *FLT3* ITD samples showing higher and more homogeneous levels of total induced apoptosis as compared to *FLT3* WT samples was not significant in the training study, likely due to the low N of *FLT3* ITD samples with high mutational load, the difference in variance between *FLT3* subgroups was statistically significant in the verification study (Levene's test P = 0.011 or 0.010 for Ara-C/dauno and etoposide).

#### Principal Component Analysis (PCA) demonstrates homogeneity of *FLT3* ITD AML across multiple pathways

**Figure 4:**
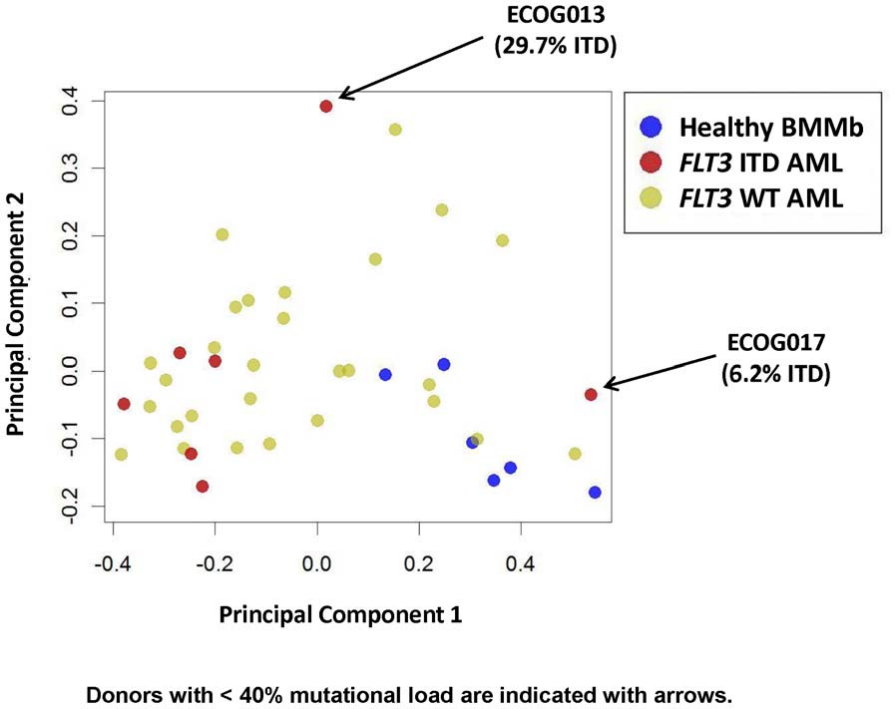
PCA pathway analysis of *FLT3* ITD AML samples and healthy BMMb compared to *FLT3* WT AML. PCA analysis of FLT3L-induced signaling (PC 1) and AraC/Daunorubicin-induced apoptosis measured by cPARP (PC 2) in healthy BMMb (blue dots), *FLT3* ITD (red dots) and *FLT3* WT (green dots). Donors with low *FLT3* ITD mutational load (<40%) are indicated by arrows.

PCA was used to combine information across multiple nodes, i.e. FLT3L-mediated induction of p-AKT, p-S6, p-ERK, and p-STAT5, IL-27-mediated induction of p-STAT1, p-STAT3 and p-STAT5, and etoposide-induced p-Chk2 and cPARP. Inspection of the first two principal components showed distinct signaling patterns among the three groups of samples: *FLT3* ITD AML, *FLT3* WT AML and healthy BMMbs (Fig. 4). Both healthy BMMb samples and *FLT3* ITD samples (with the exception of samples with mutation load <40%) formed tight but distinct clusters, suggesting tightly regulated, though discrete, signaling across these pathways. The *FLT3* WT AML samples, on the other hand, spanned a wide range of signaling and did not display distinct clusters, overlapping both the signaling patterns of the healthy BMMb and *FLT3* ITD samples.

Due to the small number of *FLT3* ITD patients in the training study, association with clinical outcomes could not be performed in this set.

### Verification Study

#### Primary Objective: Verification of decrease in range of FLT3L-induced signaling with increasing mutational load among *FLT3* ITD samples

**Figure 5:**
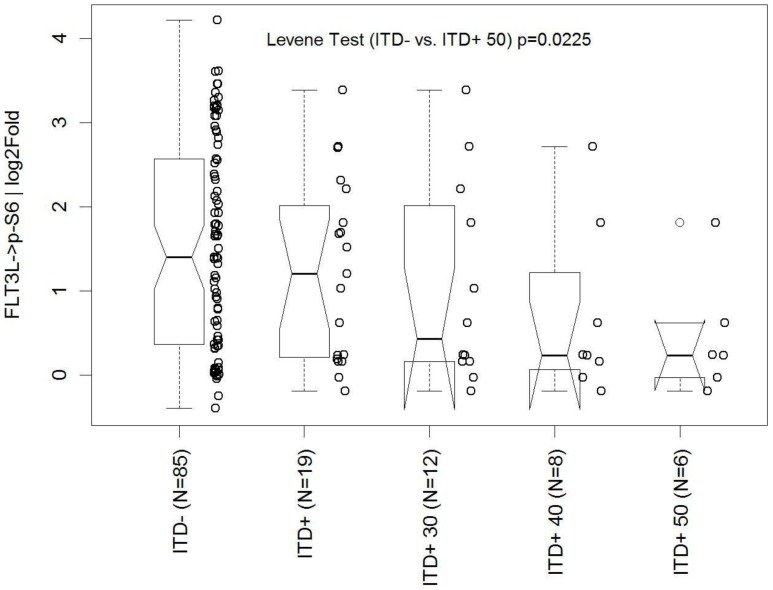
Comparison of *FLT3* ITD signaling versus *FLT3* WT signaling. Box and whisker plots of FLT3L-induced p-S6 with the log_2_fold metric in increasing mutational load (ITD−[*FLT3* WT] = 0%, ITD+[*FLT3* ITD]≥0%, ITD+30≥30%, ITD+40≥40%, ITD+50≥50% ITD, respectively). This is the primary objective analysis.

In order to verify the finding that signaling was more homogeneous in *FLT3* ITD than *FLT3* WT AML, we chose to analyze the variance of FLT3L-induced p-S6 signaling as the primary study objective, a common and relevant downstream readout in the FLT3 receptor signaling pathway. Sub-groups were defined based on the *FLT3* ITD mutational load: those with 0% ITD (*FLT3* WT, N = 85), those with measureable *FLT3* ITD levels (*FLT3* ITD+, N = 19), and inside the latter group, those with ≥30% (*FLT3* ITD+ 30, N = 12), ≥40% (*FLT3* ITD+ 40, N = 8) and ≥50% (*FLT3* ITD+ 50, N = 6) mutational load. Variance of signaling was pre-specified to be largest in the *FLT3* WT group followed in order by *FLT3* ITD+, *FLT3* ITD+ 30, *FLT3* ITD+ 40, and least in *FLT3* ITD+ 50. After unblinding clinical and mutational data, the results confirmed that in the verification sample set the FLT3L-induced p-S6 responses were muted and more homogeneous in *FLT3* ITD AML samples with high mutational load compared to *FLT3* WT AML (Fig. 5). Specifically, variance was largest in the *FLT3* WT group followed by the four defined *FLT3* ITD*+* sub-groups in increasing order of mutational load. Variance in the *FLT3* WT group was significantly (Levene Test p = 0.023) different from the *FLT3* ITD+ 50 group and marginally (Levene Test p = 0.097) different from the *FLT3* ITD+ 40 group (Fig. 5).

#### Secondary Objective: Association of SCNP node-metrics with DFS in patients with complete response

**Figure 6:**
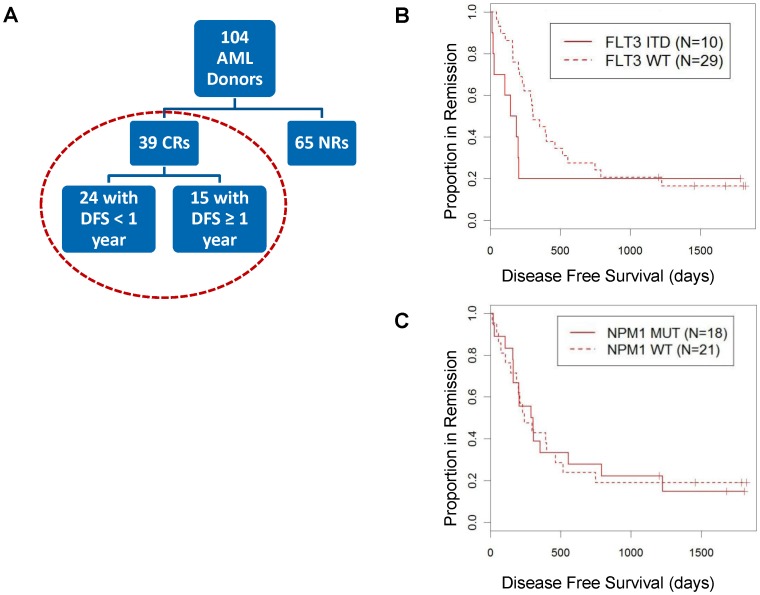
Association of *FLT3* ITD and *NPM1* mutation with DFS. (A) Patient cohort used for DFS modeling. (B) Cox-proportional hazards model for DFS using *FLT3* mutation data log h(t) = β_0_+β_1_*FLT3 ITD. Probability of DFS versus days of complete disease response (CR) for *FLT3* ITD AML samples (solid line) and *FLT3* WT samples (dotted line). (C) Cox-proportional hazards model for DFS using *NPM1* data log h(t) = β_0_+β_1_*NPM1 mutated. Probability of DFS versus days of complete disease response (CR) for *NPM1*-mutated AML samples (solid line) and *NPM1* WT samples (dotted line).

In the verification study, 65 patients had disease that was unresponsive (NR), while 39 patients experienced CR after induction chemotherapy; 37% of these CRs remained disease-free for at least one year (Fig. 6A and Table 2). The SCNP node-metric data from CR patients were combined with *FLT3* ITD and *NPM1* mutational status to identify associations between SCNP node-metrics and DFS using Cox-proportional hazards regression modeling. In this sample set, cytogenetics and gender were associated with DFS (p = 0.015 and 0.019, respectively, Table 3). *FLT3* and *NPM1* mutational status, age and secondary AML were not statistically significantly associated with DFS (Table 3 and Fig. 6B, 6C), although samples carrying *FLT3* ITD showed the expected trend of separation of survival curves (Fig. 6B).

**Table 2:** Clinical outcomes: verification cohort.

Variable	Range	*FLT3* ITD−	*FLT3* ITD+	All	*P*
Induction response	NR	56	9	65	0.19
	CR	29	10	39	
Post Remission Response (CR Patients)	Relapse or died within 1 year	16	8	24	0.20
	Continued CR for 1 year or longer	13	2	15	

Fisher's exact test was applied to categorical variables.

NR indicates non-responder; and CR, complete responder.

**Table 3:** Clinical characteristics: verification cohort (CR only).

Variable	Categories	DFS Less than 1 year n = 24	DFS 1 year or greater n = 15	All CR n = 39	*P*
Age (years)	Median	65.5	69	68	0.455
	Range	60–79	62–78	60–79	
Sex	Male	11	3	14	0.019
	Female	13	12	25	
Cytogenetics*	Favorable	1	1	2	0.015
	Intermediate/Missing	18	14	32	
	Unfavorable	5	0	5	
Race	White	23	15	38	0.231
	Black	1	0	1	
Secondary AML	De novo	19	11	30	0.112
	Secondary	4	4	8	
	RAEB-t	1	0	1	
	RAEB	0	0	0	
WBC	30 000 or less	15	11	26	0.592
	Greater than 30 000	9	4	13	
	Median (×10∧3)	15.05	9.0	14.65	0.254
	Range (×10∧3)	1.6–97.7	1.4–53.6	1.4–97.7	
*NPM1* mutated	Yes	12	6	18	0.982
	No	12	9	21	
*FLT3* ITD mutated	Yes	8	2	10	0.200
	No	16	13	29	

Modeling of DFS was performed only among patients who achieved CR to induction therapy. Each of the clinical co-variates, demographic characteristics and molecular characterics was tested for association with DFS using logrank test.

Logrank test was applied to compute p-values.

* Cytogenetics was coded as continuous variable: Favorable = 1, Intermediate/Unknown = 2, Unfavorable = 3.

DFS indicates disease free survival; CR, complete responder; WBC, white blood cell; *NPM1* nucleophosmin 1; RAEB-t, refractory anemia with excess blasts in transformation; and RAEB, refractory anemia with excess blasts.

#### Accuracy of model for DFS improves when SCNP data and molecular characterization are combined

**Figure 7:**
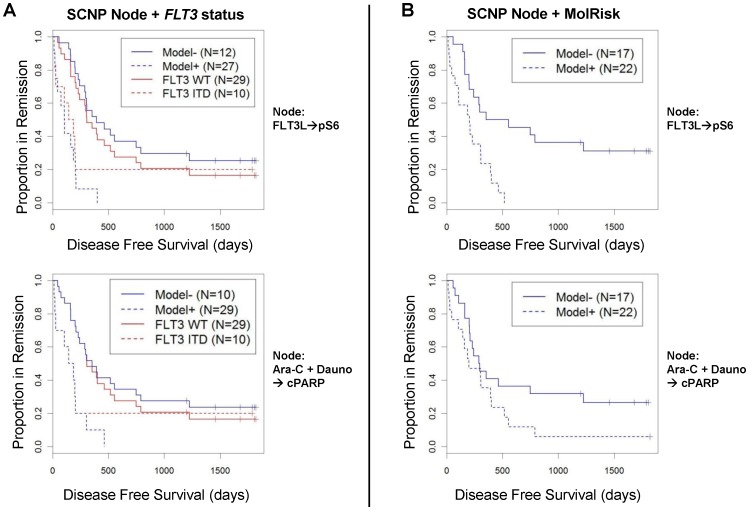
SCNP models compared to *FLT3* mutational status or molecular risk groups in modeling DFS. (A) Models for DFS based on SCNP readouts and *FLT3* mutational status allow for better separation of patients compared to modeling based on *FLT3* mutational status alone. The DFS of patients modeled based on SCNP readouts alone vs. combined with *FLT3* mutational status is shown by the blue lines (Model− vs. Model+) and DFS modeled based on *FLT3* mutational status only is shown by the red lines (*FLT3* WT vs. *FLT3* ITD). The SCNP model in the upper panel (blue lines) incorporates the SCNP node FLT3L→pS6 and in the lower panel incorporates the SCNP node Ara-C/Dauno→cPARP. (B) Models for DFS based on SCNP read outs and molecular risk group allow for clear separation of two patient groups. The DFS of patients modeled based on SCNP readouts alone vs. combined with molecular risk groups (3 groups) is shown by the blue lines (Model− vs. Model+). DFS modeled based only on molecular risk groups is not shown since it provides prediction into 3 groups. The SCNP model in the upper panel incorporates the SCNP node FLT3L→pS6 and in the lower panel the SCNP node Ara-C/Dauno→cPARP.

We first examined whether combining signaling nodes with *FLT3* mutational status improved DFS modeling. Given the small size of the data set, the complexity of the models was limited. Each regression model included a single node-metric term, *FLT3* ITD status, and an interaction term between the node-metric and mutational status. Inclusion of the interaction term was motivated by the difference in range of signaling for a majority of the nodes between *FLT3* ITD and *FLT3* WT samples. Nine of 11 nodes examined were identified to have a significant association (p_Model_≤0.05) with DFS when both node and *FLT3* mutational status were considered (Table 4). Specifically, measurements of FLT3L-induced phosphorylation of S6, PMA-mediated phosphorylation of ERK and S6, Ara-C/Dauno-induced apoptosis, and etoposide-induced apoptosis, each individually, added to *FLT3* mutational status and increased the accuracy of DFS modeling (Table 4, Fig. 7A). These SCNP nodes remained significant (p_Model_≤0.05) when tested with *FLT3* and *NPM1* mutational status together (i.e. molecular classification) as shown in Table 5 and Fig. 7B.

**Table 4:** Nodes in combination with *FLT3* mutation improve DFS modeling.

Population	Modulator	Modulation time, minutes	Antibody	Metric	*P* model* ‡	*P* node†	*P FLT3* MUT†	*P* interaction term† ∥
Leukemic§	FLT3L	10	p-S6	log_2_ fold	0.038	0.034	0.736	0.141
	FLT3L	15	p-S6	log_2_ fold	0.009	0.010	0.805	0.077
	G-CSF	15	p-STAT3	log_2_ fold	0.147	0.090	0.714	0.169
	PMA	15	p-ERK	log_2_ fold	0.004	0.005	0.252	0.019
	PMA	15	p-S6	log_2_ fold	0.002	0.003	0.302	0.009
	SCF	15	p-S6	log_2_ fold	0.030	0.020	0.899	0.089
Leukemic	AraC+Dauno	1440	CD34	U_u_	0.005	0.006	0.064	0.013
	AraC+Dauno	1440	cPARP	U_u_	0.001	0.002	0.007	0.002
	AraC+Dauno	1440	p-Chk2	U_u_	0.036	0.024	0.064	0.034
	Etoposide	1440	cPARP	U_u_	0.012	0.011	0.025	0.010
	Etoposide	1440	p-Chk2	U_u_	0.205	0.157	0.263	0.196

The table displays the *p*-values for the models (*P* model) as well as the components: node (*P* node), *FLT3* ITD status (*P FLT3* MUT), and the interaction term (*P* interaction term).

p-S6 indicates phosphorylated S6 ribosomal protein; G.CSF, granulocyte colony-stimulating factor; p-STAT3, phosphorylated signal transducer and activator of transcription 3; PMA, phorbol myristate acetate; p-ERK, phosphorylated endoplasmic reticulum kinase; SCF, Skp, Cullin, F-box containing complex; CD34, cluster of differentiation 34; cPARP, cleaved poly(ADP-ribose) polymerase; and p-Chk2, phosphorylated checkpoint 2 protein kinase.

Sample size n = 39 for each row.

* Wald test used.

† t-test (H0:Slope = 0). Significant p-value suggests influence of model component on hazard ratio.

‡ Log of hazard ratio fit of mutation data plus node with interaction term using Cox Proportional -hazards regression model: log h(t) = β_0_+β_1_*FLT3 ITD+β_2_*node+β_2_*node*FLT3 ITD.

§ Signaling examined in non-apoptotic leukemic cells (cPARP negative).

∥ Interaction term included in model to evaluate simultaneous influence of mutation and node on hazard ratio.

**Table 5:** Nodes in combination with molecular characterization improve DFS modeling.

Population	Modulator	Modulation time, minutes	Antibody	Metric	*P* Model* ‡	*P* Node†	*P* MolChar†	*P* interaction term† ∥
Leukemic§	FLT3L	10	p-S6	log_2_ fold	0.027	0.090	0.191	0.025
	FLT3L	15	p-S6	log_2_ fold	0.007	0.059	0.242	0.012
	G-CSF	15	p-STAT3	log_2_ fold	0.108	0.158	0.681	0.054
	PMA	15	p-ERK	log_2_ fold	0.023	0.083	0.134	0.021
	PMA	15	p-S6	log_2_ fold	0.003	0.015	0.096	0.002
	SCF	15	p-S6	log_2_ fold	0.038	0.195	0.519	0.042
Leukemic	AraC+Dauno	1440	CD34	U_u_	0.004	0.008	0.006	0.001
	AraC+Dauno	1440	cPARP	U_u_	0.018	0.032	0.012	0.006
	AraC+Dauno	1440	p-Chk2	U_u_	0.068	0.055	0.029	0.019
	Etopo	1440	cPARP	U_u_	0.030	0.045	0.018	0.009
	Etopo	1440	p-Chk2	U_u_	0.132	0.098	0.057	0.042

The table displays the *p*-values for the models (*P* Model) as well as the components: node (*P* Node), molecular characterization (*P* MolChar), and the interaction term (*P* interaction term).

p-S6 indicates phosphorylated S6 ribosomal protein; G.CSF, granulocyte colony-stimulating factor; p-STAT3, phosphorylated signal transducer and activator of transcription 3; PMA, phorbol myristate acetate; p-ERK, phosphorylated endoplasmic reticulum kinase; SCF, Skp, Cullin, and F-box containing complex; CD34, cluster of differentiation 34; cPARP, cleaved poly(ADP-ribose) polymerase; and p-Chk2, phosphorylated checkpoint 2 protein kinase.

Sample size n = 39 for each row

* Wald test used.

† t-test (H0:Slope = 0). Significant p-value suggests influence of model component on hazard ratio.

‡ Log of hazard ratio fit of MolChar plus node with interaction term using Cox Proportional -hazards regression: log h(t) = β_0_+β_1_*FLT3 ITD+β_2_*node+β_2_*node*FLT3 ITD.

§ Signaling examined in non-apoptotic Leukemic cells (cPARP negative).

∥ Interaction term included in model to evaluate simultaneous influence of MolChar and node on hazard ratio.

#### SCNP nodes likely contribute unique information for the prediction of DFS in *FLT3* WT AML

**Figure 8:**
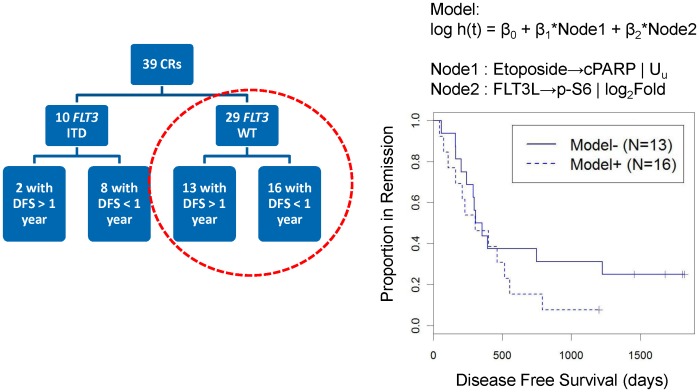
Multivariate model in *FLT3* WT AML donors using combination of SCNP nodes: association with DFS. Association of SCNP readouts from apoptosis (Etoposide→cPARP | U_u_) and proliferation (FLT3L→p-S6 | Log_2_Fold) pathways with DFS in a multivariate model among the patients with AML and *FLT3* WT disease (n = 29).

FLT3L signaling nodes and apoptosis nodes were both associated with DFS when controlled for *FLT3* mutational status. To assess whether SCNP nodes provided independent information in improving the accuracy of modeling DFS, additional analyses were performed. Specifically, focusing on the *FLT3* WT patients (Fig. 8), (a group of patients with an unmet clinical need for risk of relapse markers since few markers have been validated to associate with DFS in this patient group), the apoptosis node (Etoposide→cPARP using U_u_ metric) was combined with the signaling node (FLT3L→pS6 using log_2_(Fold) metric) using a Cox-proportional hazard regression model for DFS. This model revealed a significant association of both nodes with DFS (Table 6 and Fig. 8). Furthermore, the association between SCNP nodes and DFS remained significant (p_Model_<0.05) when accounting for the presence of *NPM1* mutation in the *FLT3* WT group (Table S2). In addition, the SCNP nodes remained significant (p_Model_<0.05) (Table S2) after controlling for cytogenetic risk.

**Table 6:** SCNP nodes likely contribute unique data for the prediction of DFS in *FLT3* WT AML.

Model	Node1	Node2	*P* Model*	*P* Node1†	*P* Node2†
#1	AraC+Daunorubicin→cPARP | U_u_	FLT3L→p-S6 | log_2_ fold	0.046	0.033	0.034
#2	Etoposide→cPARP | U_u_	FLT3L→p-S6 | log_2_ fold	0.038	0.037	0.022

log h(t) = β_0_+β_1_*Node1+β_2_*Node2.

Sample size n = 29 for each row.

* Wald test used.

† t-test(H0:Slope = 0). Significant p-value suggests influence of model component on hazard ratio.

## Discussion

Herein, SCNP was used to functionally characterize FLT3 signaling in patients age 60 years or older with AML. In two sequential studies, the homogeneity of FLT3 pathway signaling abnormalities observed in *FLT3* ITD AML samples (compared to normal BM and *FLT3* WT AML) was verified and shown to be correlated with *FLT3* ITD mutational load [27]. Furthermore, the combination of SCNP data with *FLT3* and *NPM1* mutational status improved the accuracy of modeling DFS over that provided by mutation status alone. While the relatively small number of samples analyzed in this hypothesis generating study will necesitate further study in larger sample sets, these findings demonstrate that SCNP provides insights into critical signaling pathways of individual AML cases, information which is potentially clinically relevant. Functional data such as this could ultimately be used to inform post-remission therapy selection and aid in the selection of patients for treatment with inhibitors of signaling pathways.

Ligand binding to the FLT3 receptor initiates a signaling cascade that results in the differential activation of RAS/RAF/MAPK, PI3 kinase, and/or STAT pathways in AML [19], [20], [21], [22], [23], [24], [25], [26]. In the current study, although expression levels of FLT3 receptors were similar in the two groups, *FLT3* ITD samples displayed a blunted response to FLT3L modulation (as measured by intracellular levels of p-ERK, p-AKT, p-STAT5 and p-S6) compared to their *FLT3* WT counterparts. These data confirm prior findings [27] and suggest FLT3L-independent signaling in *FLT3* ITD AML.

*FLT3* ITD samples were more sensitive to etoposide-induced *in vitro* apoptosis than *FLT3* WT samples. These data indicate the presence of an intact apoptotic machinery in *FLT3* ITD samples and suggest their increased *in vitro* susceptibility to continuous exposure of apoptosis-inducing drugs. While these data may seem counter-intuitive based on the poor survival prognosis associated with *FLT3* ITD AML, *FLT3* ITD has been reported to be associated with increased reactive oxygen species and with susceptibility to double-stranded DNA breaks with inaccurate but permissive repair [53], [54]. These impaired repair mechanisms and resultant genomic instability lead to increased apoptosis in the majority of cells but allow for increased survival of *FLT3* ITD myeloblasts which eventually cause disease relapse.

The variance in signaling was observed to be the largest in the *FLT3* WT group followed by *FLT3* ITD AML in increasing order of mutational load. The impact of *FLT3* mutational load has been evaluated in several studies but has not yet been standardized for application in clinical practice. A high ratio of the ITD allele compared to the WT allele or a high percentage of *FLT3* ITD mutation confers unfavorable prognosis, and multivariate analysis has shown allelic burden to be an independent prognostic factor for worse overall survival (OS) and DFS [2], [4], [12], [13]. Furthermore, *FLT3* ITD*/FLT3* WT ratios of 0.5 or higher in the context of mutated *NPM1* have an adverse impact on both event-free survival and OS [13]. Of note, while the thresholds used for the calculation of the impact on survival varied in each of the studies [2], [4], [12], our data shows homogeneity of signaling in AML samples starting with ∼40% *FLT3* ITD (Fig. 2), a value currently used to define high risk status in pediatric AML by the Childrens Oncology Group [55].

PCA analysis that combined information across multiple SCNP nodes showed distinct clusters in healthy BMMb and *FLT3* ITD AML (with mutation load >40%), suggesting that these cell types have tightly regulated distinct signaling pathways. Conversely, *FLT3* WT AML samples demonstrated diffusely scattered heterogeneous signaling patterns. These data suggest that prognostication based on similarities in FLT3 receptor signaling may be equally, and if used in modeling, potentially even more informative than molecular characterization and mutational load alone.

Finally, the observation that SNCP nodes from multiple pathways were found to be independent and yet significantly associated with DFS in the subgroup of patients lacking *FLT3* receptor and *NPM1* mutations (a subset of patients in need of markers associated with disease relapse) is of note and warrants further investigation in a larger patient cohort. In summary, this work demonstrates the potential utility of combining SCNP measurements of disease-relevant signal transduction pathways with molecular characterization to yield improved accuracy of DFS predictions in AML samples compared to predictions from molecular characterization alone. These results emphasize the value of comprehensive functional assessment of biologically relevant signaling pathways in AML as a basis for the development of highly predictive tests for guidance of post-remission therapy, including treatment with agents interfering with the FLT3 signaling pathway.

## Supporting Information

Figure S1 **Metrics Overview.** (A) Overview of SCNP metrics based on ERF values. (B) The Uu metric compares the ERF values of the modulated and unmodulated wells that has been scaled to the unit interval (0,1). Ua is the same as the Uu metric except for the autofluoresence control is used as the reference instead of the unmodulated well.
(TIFF)

Table S1 **List of experimental conditions and reagents used.** (**A**) List of modulators and technical conditions used. (**B**).List of antibodies and reagents used.
(TIFF)

Table S2 ***NPM1*** **mutational status does not account for association between SCNP nodes and DFS among** ***FLT3*** **WT donors.**
(TIFF)
