# Supplementary figures and images for: Functional Pathway Analysis Using SCNP of FLT3 Receptor Pathway Deregulation in AML Provides Prognostic Information Independent from Mutational Status
Source: PLoS One. 2013 Feb 19;8(2):e56714. doi: 10.1371/journal.pone.0056714 (PMC3576376; doi:10.1371/journal.pone.0056714)

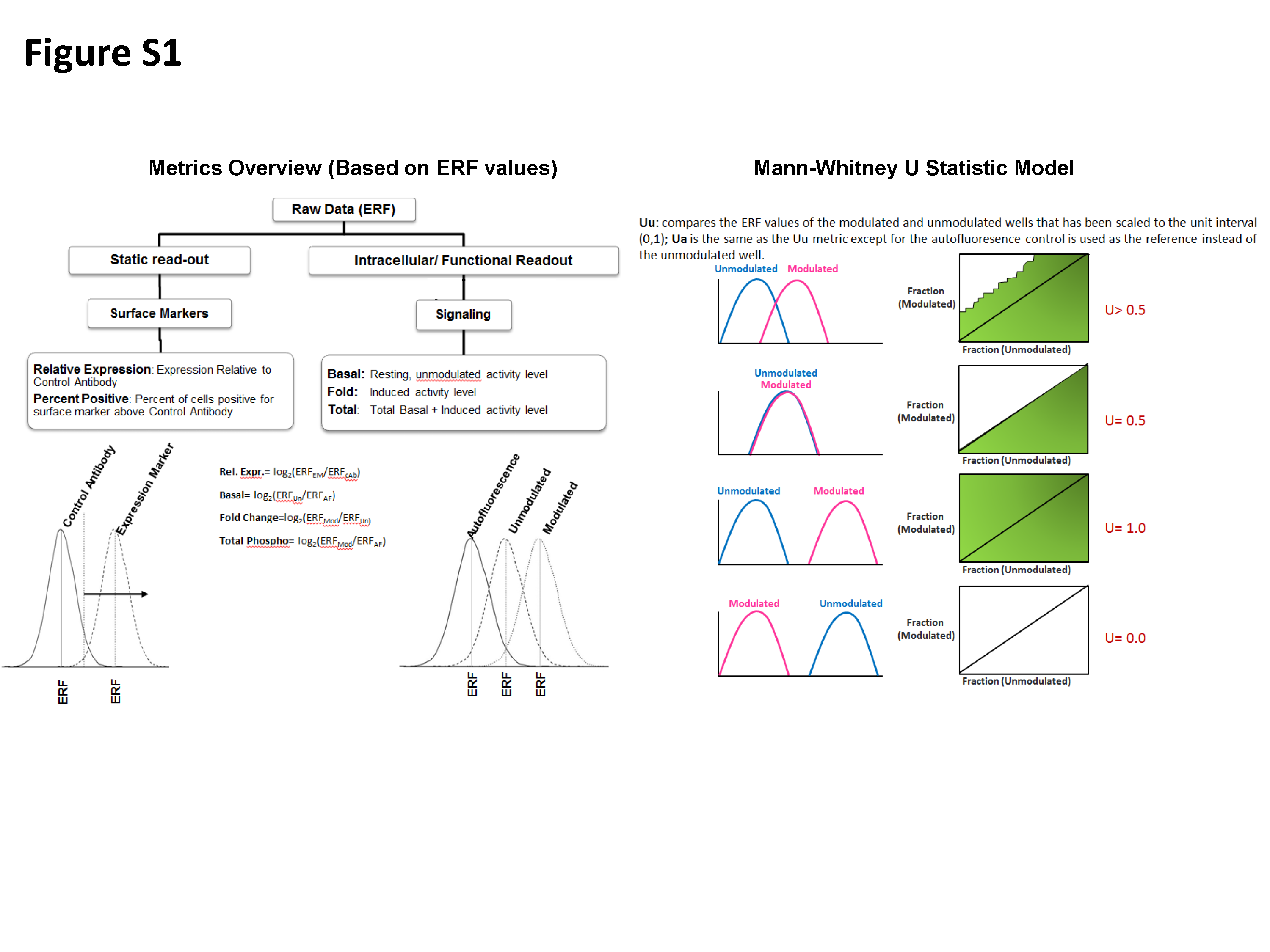

Supplement: Figure S1 — Metrics Overview. (A) Overview of SCNP metrics based on ERF values. (B) The Uu metric compares the ERF values of the modulated and unmodulated wells that has been scaled to the unit interval (0,1). Ua is the same as the Uu metric except for the autofluoresence control is used as the reference instead of the unmodulated well. (TIFF) [file pone.0056714.s001.tiff]

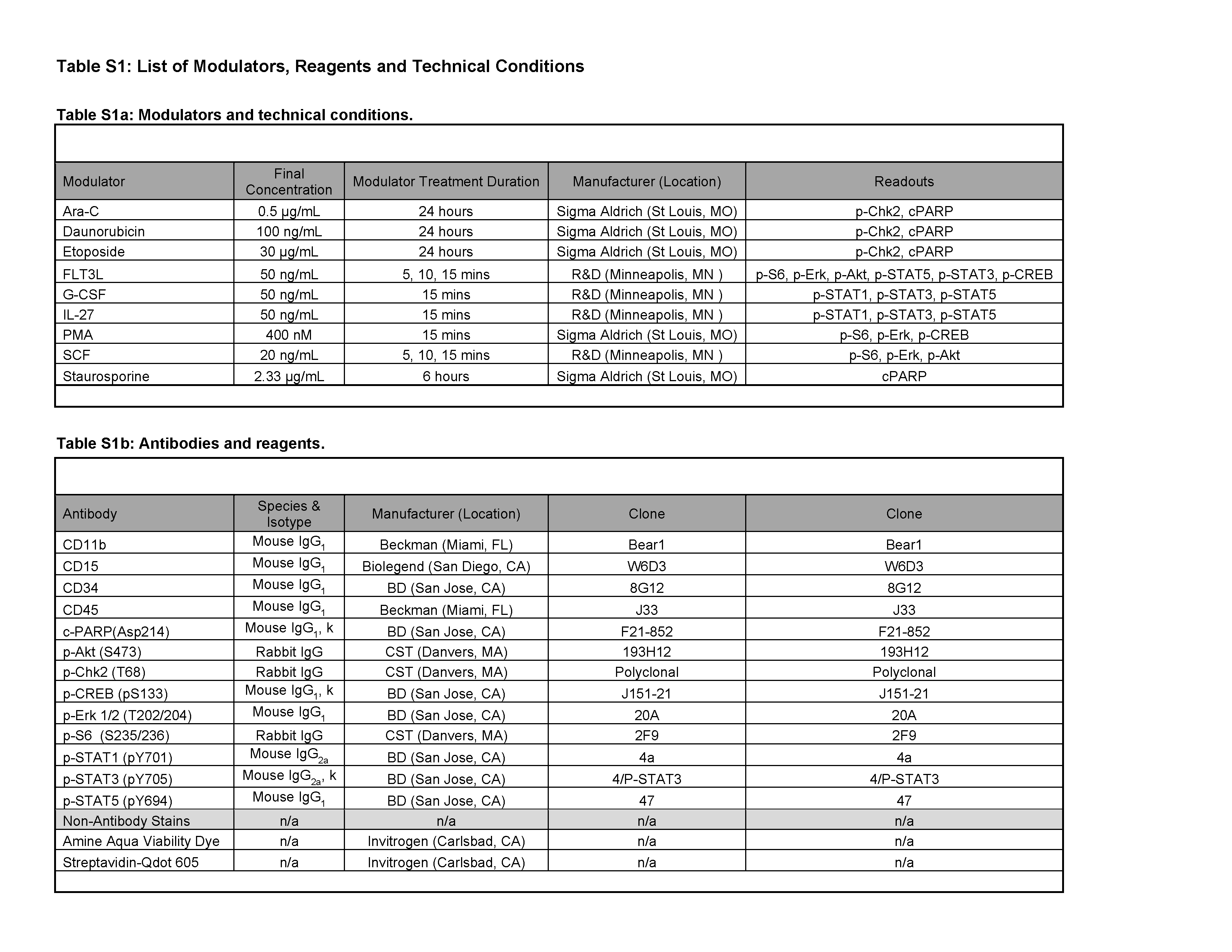

Supplement: Table S1 — List of experimental conditions and reagents used. (A) List of modulators and technical conditions used. (B).List of antibodies and reagents used. (TIFF) [file pone.0056714.s002.tiff]

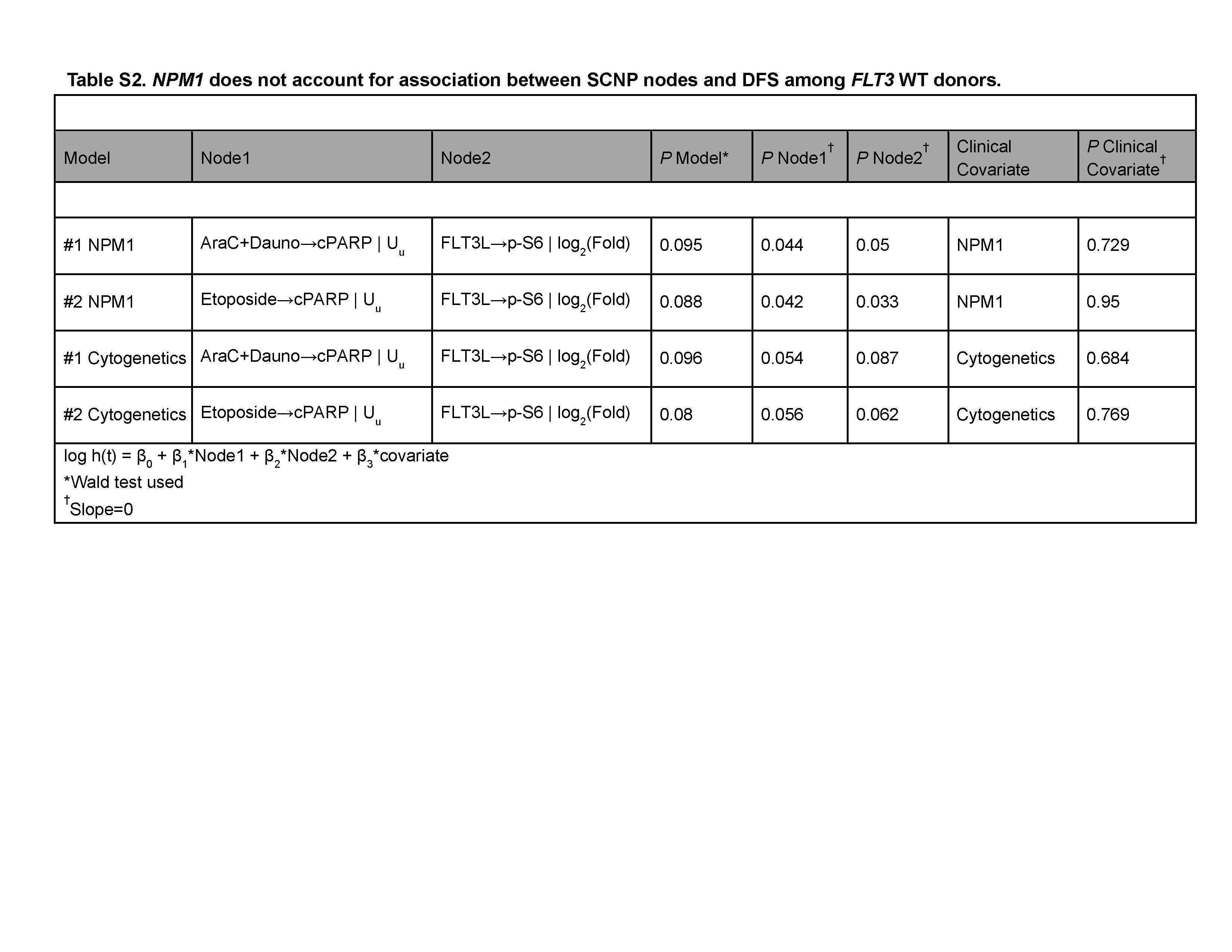

Supplement: Table S2 — NPM1 mutational status does not account for association between SCNP nodes and DFS among FLT3 WT donors. (TIFF) [file pone.0056714.s003.tiff]
